# Effects of the COVID-19 pandemic in higher education: A data driven analysis for the knowledge acquisition process

**DOI:** 10.1371/journal.pone.0274039

**Published:** 2022-09-07

**Authors:** Fátima Velásquez-Rojas, Jesus E. Fajardo, Daniela Zacharías, María Fabiana Laguna

**Affiliations:** 1 Instituto de Física de Líquidos y Sistemas Biológicos (UNLP-CONICET), La Plata, Argentina; 2 Departamento de Ciencias Básicas, Facultad de Ingeniería, Universidad Nacional de La Plata (UNLP), La Plata, Argentina; 3 Instituto de Física Interdisciplinar y Sistemas Complejos IFISC (CSIC-UIB), Campus UIB, Palma de Mallorca, Spain; 4 Departamento de Física Médica, Centro Atómico Bariloche, CONICET, CNEA, Bariloche, Argentina; 5 Departamento de Estadística, Centro Regional Universitario Bariloche (CRUB) Universidad Nacional del Comahue (UNCOMA), Neuquén, Argentina; 6 División Física Estadística e Interdisciplinaria, Centro Atómico Bariloche and CONICET, Bariloche, Argentina; 7 Profesorado en Física, Universidad Nacional de Río Negro (UNRN), Bariloche, Argentina; China University of Mining and Technology, CHINA

## Abstract

The COVID-19 pandemic abruptly changed the classroom context and presented enormous challenges for all actors in the educational process, who had to overcome multiple difficulties and incorporate new strategies and tools to construct new knowledge. In this work we analyze how student performance was affected, for a particular case of higher education in La Plata, Argentina. We developed an analytical model for the knowledge acquisition process, based on a series of surveys and information on academic performance in both contexts: face-to-face (before the onset of the pandemic) and virtual (during confinement) with 173 students during 2019 and 2020. The information collected allowed us to construct an adequate representation of the process that takes into account the main contributions common to all individuals. We analyzed the significance of the model by means of Artificial Neural Networks and a Multiple Linear Regression Method. We found that the virtual context produced a decrease in motivation to learn. Moreover, the emerging network of contacts built from the interaction between peers reveals different structures in both contexts. In all cases, interaction with teachers turned out to be of the utmost importance in the process of acquiring knowledge. Our results indicate that this process was also strongly influenced by the availability of resources of each student. This reflects the reality of a developing country, which experienced prolonged isolation, giving way to a particular learning context in which we were able to identify key factors that could guide the design of strategies in similar scenarios.

## Introduction

The process of acquiring knowledge is one of the most complex for the human being since it involves individual and social processes that have been studied by various epistemological currents [1]. The educational context where this process is developed is of great relevance since it represents the meeting space between teachers and students, in which a fundamental part of the construction of new knowledge occurs.

The COVID-19 pandemic abruptly changed this context with classroom closures of unprecedented extent and duration, disrupting conventional education in schools and universities around the world. Such measures were an extension of the isolation established in many countries to mitigate the effects of COVID-19, given that social distancing proved to be one of the most effective strategies [2–10].

The educational community as a whole made an enormous effort to quickly adapt to the distance and online learning that this lockdown brought [11], but it is no less true that students were forced to rely much more on their own resources to sustain the continuity of their learning during this period [11–13]. In the particular case of Argentina the confinement measures began on March 20, 2020, affected all educational levels and coincided with the beginning of the first semester of the academic year.

The new educational context not only brought about great challenges but was also reflected in the results obtained by the students [14–18]. The effects of the change in the learning conditions, although recent and still in process, have been analyzed from different perspectives [11–17]. A less explored methodology, which we propose to address here, is to study this problem from the point of view of complex systems, in line with what was done by some of the authors of this work just before the start of the pandemic [19]. The reason behind choosing this research design lies in the fact that the approach from this perspective allows the interactions between the individuals involved to be adequately considered when analyzing the effect of a global variable, such as the pandemic. But in addition, the usefulness of mathematical modeling to unravel the relevance of different factors that are present in the knowledge acquisition process was demonstrated in our previous work.

In [19] we developed an analytical model (the KA model) based on data from a series of surveys that are contrasted with information on academic performance of students, to analyze how the knowledge acquisition depends globally on different extrinsic and intrinsic factors. Regarding the intrinsic factors, one that contributes greatly to the acquisition of knowledge of students is motivation, and this is precisely one of the most affected by the pandemic [20]. According to the EU report [14], the closure of physical schools and the adoption of distance education can negatively affect student learning through four main channels: less time spent learning, symptoms of stress, a change in the way that students interact, and lack of motivation to learn. But, it is possible to use a model to assess the hypothesis that lack of motivation is one of the strongest negative impacts of the pandemic on students, regardless of their personal characteristics? In particular, and since the KA model was developed for a specific (face-to-face) context, the first question to be answered in this work should be whether this model is sensitive to modifications of the educational context.

On the other hand, it was already mentioned that the change in physical context affected extrinsic factors that contribute to the acquisition of knowledge, such as the interaction with peers and teachers. This interaction has been found to be essential for the development of positive self-esteem, self-confidence, and a sense of identity. In fact, there is significant evidence showing that social skills are positively associated with cognitive skills and school achievement [21, 22]. In this regard, a series of questions arise: From the perspective of the students, did the bond with teachers improve or worsen during the pandemic? Did the interaction between peers change with the change of context? What aspects of it can be measured in the new context?

Analyzing the consequences of the pandemic on the educational performance is a matter of global importance. It is well known that the distance education is essential to ensure the continuity of learning in situations in which face-to-face classes are suspended. In places where virtual and remote strategies were already becoming a reality, the change was a positive [18]. However, in other countries something as basic as Internet access is still a privilege, guaranteeing distance education cannot be taken for granted. The preparation (or lack thereof) of some countries in this area has revealed the weaknesses of educational methodologies and resources [13]. Bringing this situation to light is one more step towards fairness.

The previous statements prompt us to seek answers about how much the academic performance of students was affected by the change in the educational context caused by the pandemic. In addition, and in relation to the KA model, we would like to evaluate whether the aspects that we consider relevant have a comparable importance in the construction of knowledge, as well as the consistency of these results when comparing both scenarios.

In this new approach we adapt the analytical model presented in [19] to compare the knowledge acquisition process in two different contexts: face-to-face (before the onset of the pandemic) and virtual (during the confinement), for a particular case in higher education in Argentina. We present a study that involves 173 students and its entire evolution during 2019 and 2020 in both contexts. Furthermore, and in order to assess the relevance of the parameters we chose for our model, we apply two robust and versatile tools used in multiple applications: Artificial Neural Networks and a Multiple Linear Regression Method.

The article is organized as follows: in the Methods section we describe the participants and its educational context, the data collection and variables (which include the surveys used to construct our data-based model) and the different approaches used to fit the parameters of the model. Then, we present the main results of this work and finally, we summarize and discuss our findings.

## Methods

### Educational context

The research was carried out with several sections of students who attended the Physics II course, corresponding to the second year of Engineering careers at the Faculty of Engineering of the National University of La Plata (UNLP) [23], Argentina, during the years 2019 and 2020. The Faculty offers 13 engineering degrees, so the interest of the students in the course can vary greatly.

The complete course lasts one semester, with a workload of 8 hours per week divided into 2 theoretical-practical classes. The course consists of two parts, at the end of which a partial written test is taken with a score between 0 and 10. There are two approval regimes: direct promotion, which implies being exempt from the final test (if the average between the two partial exams is 6 or more) or promotion by final exam (if the average is between 4 and 6). Partial tests have an instance of recuperation during the semester and another at the end of it, where the student can improve any of the lower scores obtained in previous tests. This organization was also maintained during the confinement (in virtual context).

### Participants

The first part of the research was done during the two semesters of the year 2019, with four different sections in face-to-face context for a total of 81 students (50 male, 31 female). The second part was developed during the year 2020 and also involved four different sections in two semesters, for a total of 92 students (61 male, 31 female). In all cases we had access to the final grade they obtained in the course. In both contexts, we worked with 4 different sections of students for a total of 8 sections, 173 students in 2 years. The initial group of students was much larger, however there were 173 who participated in the whole process. These data are reported in the (S1 File) and has been collected with the following actions:

- It does not involve minors.
- It has been collected anonymously. Students have been identified by a numerical code, avoiding gathering of any personal information.
- Students have been informed by the lecturers that some information about their activity could be anonymously collected for statistical purposes. Authors of this study did not receive any objections.
- The tasks related to this study were completely voluntary and they did not in any form alter students’ activities, classes, or the assessment process.

Considering these circumstances, we do not need to apply for ethics approval from our university since no personal data, minors or potentially hazardous activities were involved in the study.

Besides, all teachers involved in the study (some of them also co-authors of this manuscript) who were responsible for the subject taught also gave consent to carry out the study.

We obtained verbal consent from all the participants in the study.

### Data collection and variables

We are interested in analyzing and comparing the processes observed in both contexts in terms of the KA model presented in [19]. A first step consisted in carrying out a classification such as that proposed by Bordogna and Albano [24] and which proved to be useful in our previous work. This involved separating the students into three different groups according to their final achievements *K*_*f*_, which we relate to the final grade obtained in the course. This was done as follows: (a) High-achieving (HA) students: 8 ≤ *K*_*f*_ ≤ 10, (b) Average-achieving (AA) students: 6 < *K*_*f*_ < 8 and (c) Low-achieving (LA) students: *K*_*f*_ ≤ 6. It is worth noting that students with a final grade lower than 4 are not included in this study.

In Table 1 we show the number of students who participated in the work divided according to their final achievements *K*_*f*_, that we relate to the final grade obtained in the course. Interestingly, and as we found in [19], the groups have qualitatively different characteristics regarding the relevance of the factors considered in the construction of the new knowledge, as it will be clear shortly.

**Table 1:** Number of students participating in the work, divided according to their final achievements *K*_*f*_ in both contexts.

Context	HA	AA	LA	Total
Face-to-face	13	34	34	81
Virtual	19	42	31	92
**Total**	**32**	**76**	**65**	**173**

**Fig 1:**
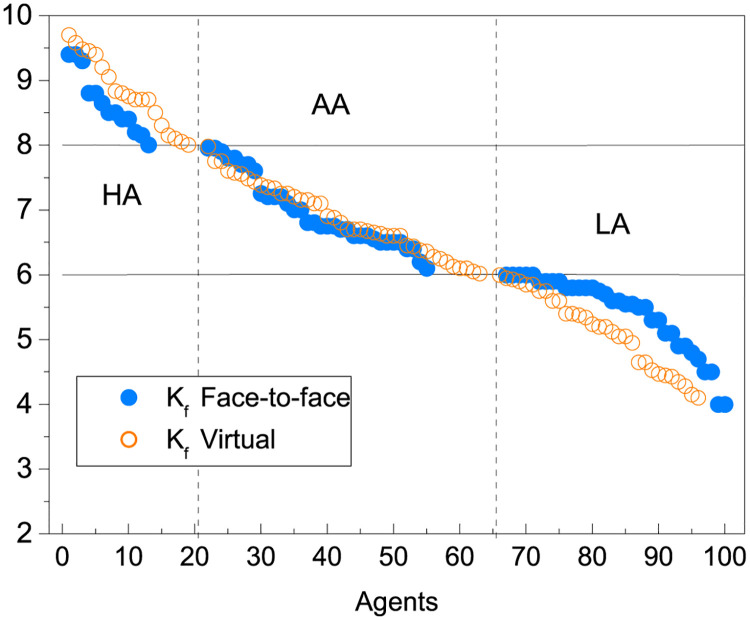
Group classification of students into three different sets according to their final grade as High-achieving (HA) students, Average-achieving (AA) students and Low-achieving (LA) students for *N* = 173.

In Fig 1 we display the final grades obtained for all students that we include in the present work. In filled symbols we plot the data in the face-to-face context and the empty symbols represent the data in the virtual context. These data provide us with the information to contrast our theoretical model. A first look at this graph reveals that the marks obtained in the two contexts were different for the HA and LA groups, while the AA group did not present differences. HA students, whose grades were higher than 8, had on average a better performance in virtual context than in face-to-face context. The opposite is seen with the Low-achievement students, LA. To analyze the possible causes of these differences is one of the main purposes of the present paper.

#### KA model for both contexts

Inspired by our previous work and in order to explicitly compare the classroom observations and surveys with the final achievement of the students, we write the final knowledge on a given topic as the sum of a set of contributing factors. For a student *i* = 1, 2, 3, …*N*, its final knowledge $K_{f}^{i}$ can be posed as:

$$\begin{array}{c} K_{f}^{i}=\beta_{M}^{X}M^{i}+\beta_{T}^{X}T^{i}+\beta_{P}^{X}P^{i} \end{array}$$ (1)

where each factor of Eq 1 was collected through surveys (of own authorship) in which we analyze their levels of: personal motivation (*M*) with questions to know experiences and expectations in relation to the course; influence of the teachers (*T*) and the influence of peers (*P*). In the next section we detail the numerical values assigned to each factor.

The coefficients $\beta_{M}^{X}$, $\beta_{T}^{X}$ and $\beta_{P}^{X}$ modulates each factor of Eq 1 and they are different according to the group of students being described, *X* = HA, AA or LA. Their values were calculated using two methods that we describe further down and that consist of neural networks and a linear regression analysis.

The values of $K_{f}^{i}$ obtained from Eq 1 were compared with the final score obtained by the students, which ranges from 0 to 10.

It is worth noting that in our study the contribution of peers to the acquisition of knowledge was gathered in two ways: the group conformation and the peer interaction itself. The group conformation includes information on the spatial distribution of the students and the formation of groups, obtained through direct observations of the classes before confinement and through questions in online surveys during confinement. An analysis of the differences in the structure of the peer network formed in each context is carried out in Fig 4 in the Results section.

During the virtual context, important and complementary information was also collected, such as resources the students had (work-space, technological equipment) and the context itself and how it was perceived. Although they are not included as terms in Eq 1, we carry out a description of the observed situation in the S2 File.

Finally, it should be noted that in our study we focus on a specific type of learning, related to scientific concepts of classical physics. While we are aware that this is not the only value learned in the classroom, we simplify the concept of knowledge to use the final grade as a concrete and quantifiable measure of the student’s performance.

#### Surveys

Here we present the surveys carried out on students during each semester of classes (Table 2). The numbers and letters in the last column correspond to the values that we assign to each of them, in order to transfer the answers to the KA model of Eq 1. The questions marked with (*) were reformulated to adapt them to the virtual context. The surveys carried out in the virtual context were delivered and completed in a digital way using Google tools, while those corresponding to the pre-confinement stage were delivered personally and were completed manually.

**Table 2:** Surveys carried out on students during the semester of classes for two years (the duration of this study). Although the surveys were broader, here we only include the questions involved in the model.

Survey	Quantities	Item	Options	Values
1 (first day of the course)	*M*	At the beginning of Physics II, what is your level of expectation?	Much	1
			Intermediate	0.5
			None	0
		Because the course:	Excites me	1
			It is a requirement	0
2 (end of the first part of the course)	*M*	So far, describe your experience in Physics II:	I really like it	1
			I like it	0.5
			It is indifferent to me	0
			I do not like it	-0.5
	*P*	At a general level, describe your way of studying:	Alone	0
			In group	0.5
3 (end of the course)	*M*	At a general level, describe your experience in Physics II:	I really like it	1
			I like it	0.5
			It is indifferent to me	0
			I do not like it	-0.5
	*T*	Were the lectures useful for you?	Yes	1
			Little	0.5
			No	0
		Was the interaction with the rest of the teaching team useful to you?	Yes	1
			Little	0.5
			No	0
	T*	Were the virtual lectures useful for you?	Yes	1
			Little	0.5
			No	0
		Was the interaction with the rest of the teaching team useful to you?	Yes	1
			Little	0.5
			No	0
	*T*	At the time of study, which activity was the most beneficial for you? (You can check several options)	Lectures	A
			Consultation hours with my group	B
			Office hours	C
			Private tutoring	D
	T*	At the time of study, which activity was the most beneficial for you? (You can check several options)	Virtual Lectures	A
			Consultation hours with my group	B
			Office virtual hours	C
			None of the above	D

The quantities evaluated more than once (as is the case of *M* or *T*) were averaged in order to have a single value for each factor. Besides, the combination of strategies for the question that measures the interaction with the teacher *T* in the third survey was given the following numerical values: ABC = AB = AC = BC = 1, A = B = 0.7, C = AD = BD = ABD = ACD = BCD = 0.5, CD = 0.3, D = 0.1 (students could mark several options). These values were given to enhance the use of the strategies provided by the specific section to which the students belonged (options A, B).

#### From surveys to KA model

We present an illustrative example of how we translate the surveys into the numerical values associated to the factors of the KA model. Table 3 is an excerpt from the data presented in the (S1 File). For each context, we indicate the student’s identification, the group to which she/he belongs, and the results obtained for each term (M, T, P) normalized to 1. These values are used in Eq 1 to estimate a value of $K_{f}^{i}$ for the student *i* = ID, assigning appropriate values to the *β* coefficients (see next section). The final grade obtained with the model, using the ANN approach for the coefficients, are included in the column labeled $K_{f}^{ANN}$, and the final grade obtained with MLR ($K_{f}^{MLR}$) in the next one. Finally, the last column shows the actual final score this student obtained (*K*_*f*_).

**Table 3:** Excerpt from the data with the surveys information.

Context	ID	Group	*M*	*T*	*P*	$K_{f}^{ANN}$	$K_{f}^{MLR}$	*K*_*f*_
Face-to-face	4	HA	1.00	0.9	0.5	8.60	9.66	9.40
	5	AA	0.75	0.9	0.5	7.19	7.17	6.50
	1	LA	0.88	1.0	0.0	5.29	6.38	5.80
Virtual	1	HA	0.75	0.9	1.0	9.54	9.08	9.70
	18	AA	0.59	0.9	1.0	8.02	6.80	6.65
	25	LA	0.44	1.0	0.5	5.30	5.37	5.85

### Proposed tools for analysis

As it was already mentioned, each of these groups has different characteristics regarding the relevance of the factors considered in the construction of Eq 1. To explicitly measure the weight of each of them we apply two different and complementary approaches: Artificial Neural Networks (ANN) and a Multiple Linear Regression Method (MLR).

In reference [25], the capability of the ANN for estimating parameters of complex nonlinear and linear problems has been shown. A single-layer perceptron (SLP) constitutes a particular case of the ANN whose output equation resembles Eq 1. This allows to cross-validate the MLR, which is the most common form of linear regression analysis to treat this kind of problem.

#### Single Layer Perceptron (SLP) network overview

**Fig 2:**
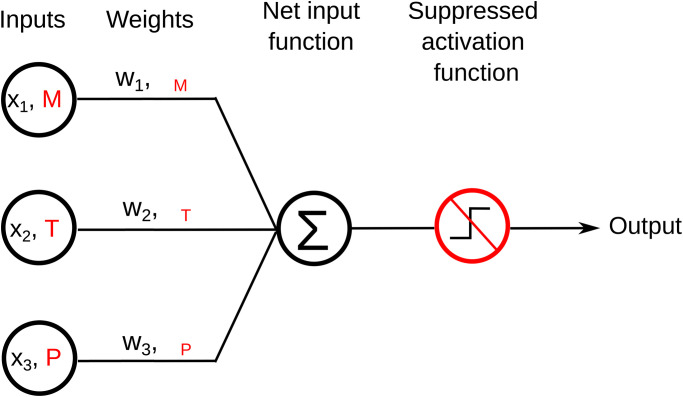
SLP ANN schematics. The usual ANN notation is in black text and in red text, the equivalent terms corresponding to this particular work are shown. (See Eq 1). The element-wise product between the inputs and the weights are added up in the “net input function” stage and suppressing the activation function, an output corresponding to the linear combination of the inputs and the weights is obtained.

To reproduce Eq 1 from an ANN architecture we employed a SLP [25]. This type of ANN constitutes a particular case of a Multilayer Perceptron (MLP) [26]. The SLP is a feedforward network of a single artificial neuron-like unit, whose *x*_*j*_ inputs (disposed akin to biologic dendrites) are multiplied by a corresponding weight *w*_*j*_ and this product is passed to a neuron-like unit where the aforementioned product is added up, as shown in Fig 2.

This product is typically passed to an activation function to mimic the activation of biological neurons, but this operation can be suppressed to get the linear combination of the inputs and its weights directly, obtaining a the neuron output:

$$\begin{array}{c} z=\sum_{j=1}^{m}x_{j}w_{j}=w^{T}x \end{array}$$ (2)

where Eq 2 corresponds to a linear combination analogous to Eq 1.

The inputs *x*_*j*_ and weights *w*_*j*_ in Eq 2 correspond, respectively to the *M*, *T*, *P* and the coefficients $\beta_{M}^{X}$, $\beta_{T}^{X}$, $\beta_{P}^{X}$ in Eq 1.

The weights (coefficients) values are obtained during the network training. In this stage the experimental data (*M*^*i*^, *T*^*i*^ and *P*^*i*^ of each student *i*) are passed to the SLP with randomly initialized weights and an output $z_{i}^{\prime }$ is calculated. Then the result is compared in each iteration to the actual *z*_*i*_ values (corresponding to each experimental $K_{f}^{i}$ of Eq 1) and a loss is calculated. As loss metric, we used the mean squared error (MSE) which is one of the commonly used in ANN, particularly in regression applications: $MSE=\frac{1}{n}\sum_{i=1}^{n}{\left(z_{i}^{\prime }-z_{i}\right)}^{2}$.

After evaluating the loss, we used the gradient descent (GD) technique to update the gradients. In short, the weights are updated through the network from *z* to *x* (right to left in Fig 2) in all the neuron-like unit in a direction opposite to that of the gradients with the rule:

$$\begin{array}{c} w≔w+\Delta w \end{array}$$

where $\Delta w_{j}=-\eta \cdot \frac{\partial J(w)}{\partial w_{j}}$, *J*(*w*) is the objective or loss function parameterized to the model parameters **w** and *η* is the learning rate, a parameter chosen between 0 and 1 (typically 0.1 is used for most applications). Once a minimum (ideally a global one) of the function *J*(*w*) has been reached and the loss is low enough, the weights vector **w** (corresponding to *β*_*M*_, *β*_*T*_, *β*_*P*_ coefficients in this particular case) is saved and the multivariate linear model is obtained.

The SLP model was implemented in the programming language Python by means of the Keras package [27].

#### Multiple Linear Regression Method

The KA model described by Eq 1 can also be approached by posing a multiple linear regression model (MLR), where the dependent variable is $K_{f}^{i}$ and can be explained by the independent variables *M*, *T* and *P*, taking into account the different groups of students (HA, AA or LA) and the context (face-to-face or virtual) to which each student belongs. Through the MLR method, the measures of the strength of the relationship between the target and predictor variables, the construction of tests of hypothesis and confidence intervals related to regression parameters are expected to be obtained. The model is expressed as:

$$\begin{array}{c} K_{f}^{i}=\beta_{M}M^{i}+\beta_{T}T^{i}+\beta_{P}P^{i}+\beta_{HA}HA^{i}+\beta_{LA}LA^{i}+\beta_{F}F^{i}+\epsilon^{i} \end{array}$$ (3)

where *i* goes from 1 to 173 (the total number of students) and *HA*, *LA* are dummy variables created to indicate the group to which the student belongs: *HA* = 1 if the student belongs to the HA group and *HA* = 0 if it does not belong to such group. In the same way, *LA* = 1 if the student belongs to the LA group and *LA* = 0 if it does not belong to the LA group. AA is considered the reference group, i.e., if a student belongs to this group, *HA* = 0 and *LA* = 0. Moreover, *F* is a dummy variable created to indicate the specific physical context to which the student belongs: *F* = 1 if the student participated in the face-to-face context and *F* = 0 if the student participated in the virtual context. Finally, *ϵ* represents the random error. We assume that the random terms *ϵ*^*i*^ have independent normal distributions with mean zero and constant variance.

Besides, *β*_*M*_, *β*_*T*_, *β*_*P*_, *β*_*HA*_, *β*_*LA*_ and *β*_*F*_ are the regression coefficients corresponding to the variables *M*, *T*, *P*, *HA*, *LA* and *F*, respectively, and they were estimated through the OLS (Ordinary Least Squares) method.

This model was fitted using the function lm() in the programming language R version 4.1.0 [28].

## Results

### Comparison between contexts

In our previous work [19], we compared the results of our KA model with the final grade that the students obtained. Looking for an answer to our main question, about how the educational context affected student performance, we first compare the general results in both, face-to-face and virtual contexts.

**Fig 3:**
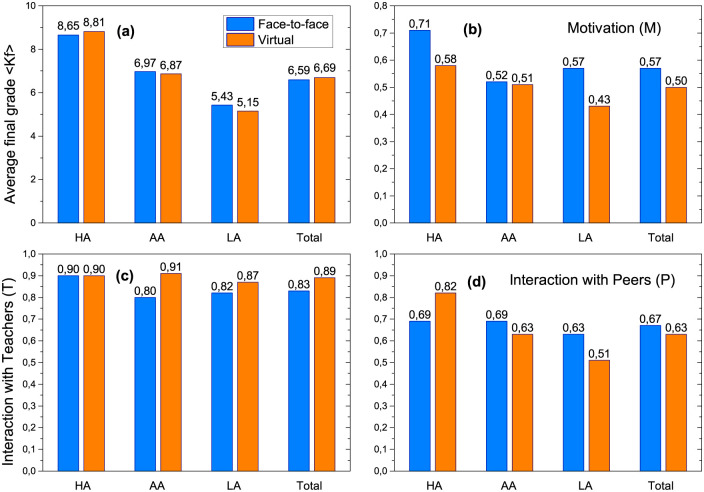
Numerical values associated with different quantities discriminated according to context, face-to-face (blue) and virtual (orange), averaged for each group of students HA, AA and LA. (a) Final grade <*K*_*f*_>, (b) motivation *M*, (c) interaction with teachers *T* and (d) interaction between peers *P*.

We proposed in Eq 1 that the final knowledge reached by a student on a given topic is mainly due to three contributing factors, the personal motivation (*M*), the influence of the teachers (*T*) and the influence of peers (*P*). In Fig 3 we show the average values of the final grade of each group, <*K*_*f*_>, together with average of the data obtained from the surveys carried out, in order to analyze and compare the differences observed with the change of context.

These results allow us to respond positively to our first question, about whether our approach is sensitive to changes in the educational context. As we can see, although the KA model was originally developed for a specific context (face to face), the values of the variables are different for both contexts.

In Fig 3(a) we show the average final grade <*K*_*f*_> for each group of students (HA, AA and LA) and in both contexts. We observe again the differences we first noticed in Fig 1, related to how the performance of each group is modified with the change of context. For HA students, <*K*_*f*_> increased during the virtual context while for AA and LA it decreased. In what follows, and to deepen the understanding of what is observed, we will analyze what was obtained for the three contributing factors (also averaged for each group), and that we plot in panels (b), (c) and (d) of Fig 3.

The values of motivation presented in Fig 3(b) reflects a widely studied aspect of the psychological impact of the pandemic on students [14, 20]. Our results clearly report the impact of the virtual context on the motivation of students, no matter the group they belong to. This fact should in itself be an alarm to build policies to support the mental health and educational success of the students at all times. If motivation dropped notably in the new virtual context, and the final knowledge is considered as the sum of several factors that contribute to the acquisition of this knowledge, then the way of interacting with peers and teachers also had to change.

The general decrease in the virtual context observed in motivation is not repeated in the other factors analyzed in this study. Fig 3(c) gives us information about the teacher’s contribution from the students’ perspective. Note that for the HA group it has the same weight in both contexts (face-to-face and virtual), while for the AA and LA groups the interaction with teachers increased in the virtual context. Generally, the teacher acts as an intermediary between the activities carried out by the students in order to assimilate the new knowledge and in this new context their presence and support (albeit virtual) was fundamental for many students.

Finally, in Fig 3(d), we can see the differences in the interaction between peers for each group of students, another issue that was affected during the pandemic.

We can see that HA’s enriched the study in groups in the virtual context in contrast to the other groups of students. We also found that the structure of the emerging contact network from peer interaction presents very different characteristics in both contexts. More details about this aspect of the problem are presented in the next subsection. The situation observed in Fig 3(d) for the interaction between peers is the one that most reflects the behavior of the general performance (Fig 3(a)), however the trend is attenuated due to what is observed in Fig 3(b) and 3(c). These results may partially respond to the change observed in the way students interact.

The aforementioned results can be summarized in Table 4 where we show the relative changes between both contexts. This quantity expresses what it was observed in Fig 3 with the raw data obtained in the surveys: A strong decrease in the motivation term for all groups of students, and different trends in the way of interacting with peers and with teachers depending on the group to which the students belong.

**Table 4:** Relative changes in % for the quantities involved in the KA model between both contexts.

	relative change (%)			
Quantities	HA	AA	LA	Total
*K*_*f*_	1.8	-1.5	-5.1	1.5
*M*	-17.5	-2.2	-23.7	-12.3
*T*	-0,1	12.8	5.9	7.1
*P*	17.8	-8.5	-19.7	-5.8

Relative change is calculated as Δ/reference, where Δ is the subtraction between the values in the virtual and face-to-face contexts, and the reference is the value in face-to-face context.

#### Networks of peer interactions

The analysis carried out around Fig 3 indicates that the change in physical context modified the way in which students interact with each other. Furthermore, in this area data was collected in different ways depending on the context. In the face-to-face context, the observations in the classroom were made in situ, with photographic records and paper surveys. During the virtual context, the surveys were digital using Google tools as mentioned above. In the latter case, no observations could be made, so the students were asked how their interaction with the group was and with whom they specifically interacted. This fact could result in a lack of information for this context. However, that was not the case, since although the information collected in both cases is not completely comparable, they suggest a change in behavior in the relationship between peers. Table 5 expresses the number of students who were observed grouped or isolated during the face-to-face classes. Likewise, for the virtual case, the number of students who affirmed to study or not in a group is reported. We find that the percentage of isolated students decreased from 37% to 26% with the change of context. Interestingly, the increase in interaction between students in the virtual context was observed to a greater or lesser extent for the three groups.

**Table 5:** Number of students who were grouped or claimed to be in a group and the isolated students for each context.

	Isolated students			In groups		
Context	HA	AA	LA	HA	AA	LA
Face-to-face	3	12	15	10	22	19
Virtual	2	11	11	14	30	22

**Fig 4:**
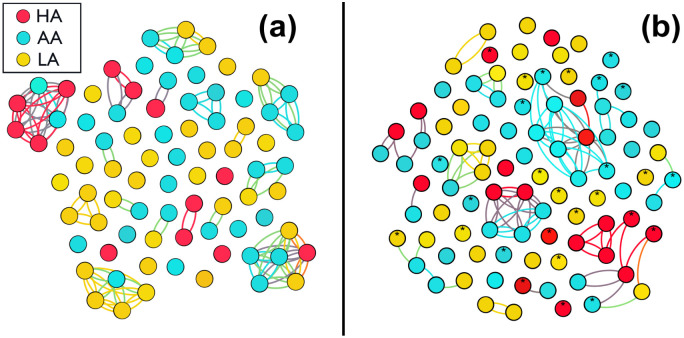
Peer interaction of different groups of students in both contexts. (a) Network scheme from classroom observations in face-to-face context where the links are reciprocal interactions. (b) Network scheme from data obtained through surveys in virtual context. The links can be or not be reciprocal interactions. The nodes marked with an asterisk represent students who claimed to interact with students from another section who did not participate in this study.

To deepen the understanding of how students modified their way of interacting, we draw in Fig 4(a) the network that represents the students before confinement (face-to-face context) for *N* = 81. As we said, the data was obtained from direct observations in the classroom, where the nodes represent the students (divided in the HA, AA and LA groups) and the links their interactions. Note that here we use double bonds, indicating a reciprocal interaction.

Besides, in Fig 4(b) we show the network that describes the students in virtual context for *N* = 92. The data come from the surveys carried out, and again the nodes represent the students divided in the groups HA, AA and LA. We use links to represent their interactions, although now they are double or single, as the responses to the surveys given by the students may or may not be reciprocal. Moreover, the nodes marked with an asterisk represent students who claimed to interact with students from another section who did not participate in this study.

A comparison between both networks indicates some similarities, such as the presence of highly connected clusters, as well as isolated students. However, the network corresponding to the virtual context has nodes that connect two different clusters, acting as “bridges”. This was not observed in the face-to-face context and could mean a new form of relationship between students. This result deepens the understanding of the effect that the pandemic has on peer relationships, and answers some of the questions asked in the introduction on this topic.

### Measure of the relevance of the terms that influence the knowledge acquisition process

A way to validate the model presented in Eq 1 is to analyze the relevance of the terms that compose it. In our previous work [19] we did it by adding coefficients to each factor of the KA model. These coefficients could be interpreted as the relative weight that each term in Eq 1 has, and were chosen so that the average value calculated with the model for each group is as close as possible to the average value of the actual final grades obtained. In order to analyze the relevance and consistency of the factors that we chose to describe the knowledge acquisition process, we now we choose two different and complementary approaches to find the weight of each term of Eq 1: Artificial Neural Networks (ANN) and a Multiple Linear Regression Method (MLR).

#### ANN approach

**Fig 5:**
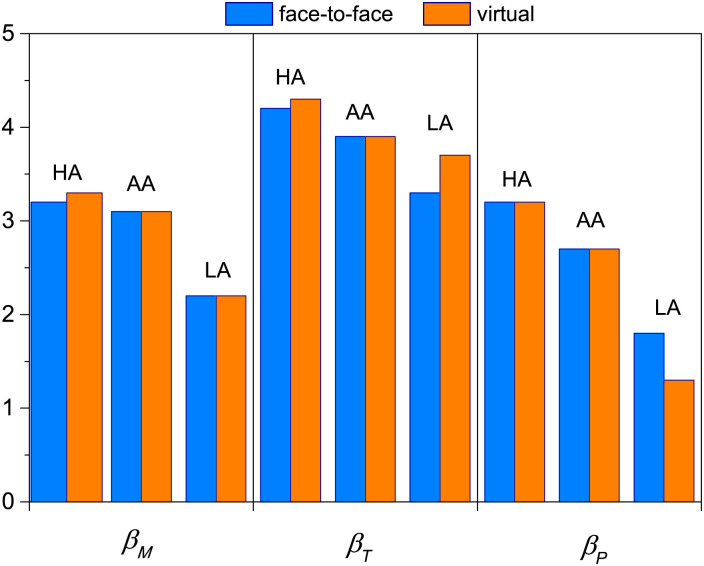
Coefficients $\beta_{M}^{X}$, $\beta_{T}^{X}$ and $\beta_{P}^{X}$ for each group of students HA, AA and LA obtained with the ANN approach. For each context these coefficients preserve generality with greater importance in the term associated with the interaction with teachers ($\beta_{T}^{X}$).

Using a SLP ANN, we were able to get the coefficients of Eq 1 for each group and the two contexts analyzed, as shown in Table 6. The values obtained indicate a generality in the weights of each factor that participates in the knowledge acquisition process, with greater prominence in the term associated to the interaction with teachers ($\beta_{T}^{X}$) for all groups. This is clearly observed in Fig 5, where a graphical representation of the coefficients is shown. These results provide robustness to the model, as the weight that each factor has in the process we are describing is independent of the context analyzed. Moreover, and as will be clear soon, the ANN predicted similar coefficients to those obtained by MLR (see next section).

**Table 6:** The coefficients $\beta_{M}^{X}$, $\beta_{T}^{X}$ and $\beta_{P}^{X}$ obtained by ANN.

	Face-to-face context			Virtual context		
	HA	AA	LA	HA	AA	LA
$\beta_{M}^{X}$	3.2	3.1	2.2	3.3	3.1	2.2
$\beta_{T}^{X}$	4.2	3.9	3.3	4.3	3.9	3.7
$\beta_{P}^{X}$	3.2	2.7	1.8	3.2	2.7	1.3

**Fig 6:**
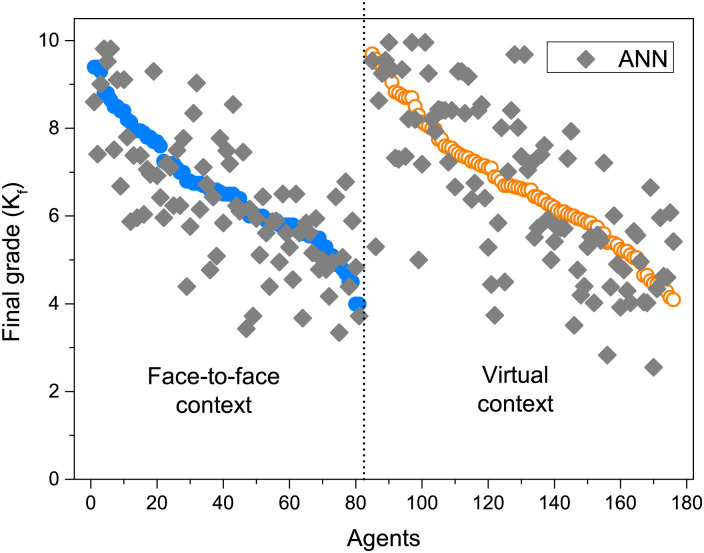
Final knowledge comparison between the actual final grade obtained for each student in both contexts (circles) and the KA Model of Eq 1 using the coefficients obtained with the ANN approach (diamonds).

Finally, in Fig 6 we present a comparison between the final grade for each student and the final knowledge obtained from Eq 1 (KA model) with the coefficients obtained with the ANN approach. The global behavior of the KA model follows the general trend of the data. The observed dispersion is due to the presence of particular cases, whose complete evolution is not captured by the model. In our previous work [19] we made an analysis of some particular cases like these.

#### MLR approach

We make use of the Multiple Linear Regression Method in order to find the weights of each contributing factor of the KA model, and compare them with the ones obtained in the previous section. The results are shown in Table 7, where we express the values for *β*, SE (standard error) and p-value for the terms of the Eq 3.

**Table 7:** Regression results of multiple linear model of Eq 3.

	*β*	SE	p-value
*M*	2.2776	0.3768	1.00e-08
*T*	4.6893	0.3063	< 2e-16
*P*	1.4660	0.2308	12.09e-09
**Student group according to their final achievements *K*_*f*_** (Reference → AA)			
*HA*	1.9131	0.2664	2.43e-11
*LA*	-1.0585	0.2136	1.81e-06
**Context** (Reference → Virtual context)			
Face-to-face context	0.7424	0.1902	0.000139
**Adjusted R-squared**			0.9664
**p-value**			< 2.2e-16

Note: the lower limit of the values for T was modified as $T_{resc}=\frac{T-0.33}{1-0.33}$ to coincide with the scales of the rest of the variables.

The p-values obtained show that all beta regression coefficients are statistically significant. Assumptions of linearity, independence, homoscedasticity and normality were checked, as well as the presence of influential values.

We find that the term with the highest weighting is the one related to the interaction with teachers ($\beta_{T}^{X}$), in accordance with what was obtained with the ANN approach.

**Fig 7:**
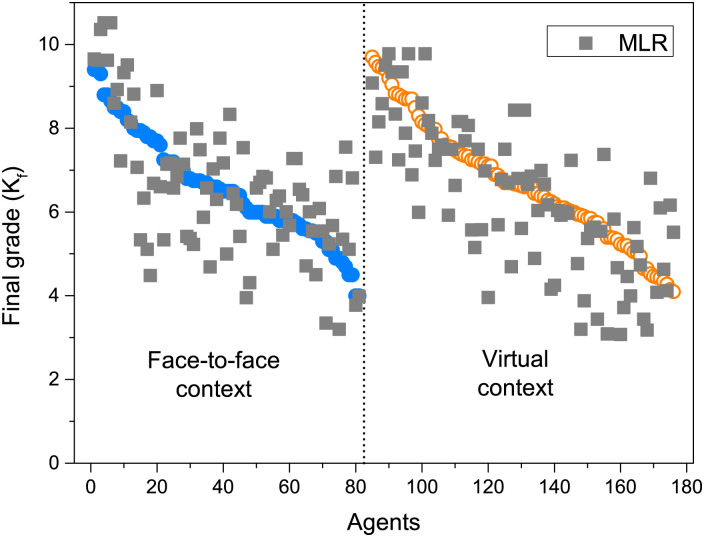
Final knowledge comparison between the actual final grade obtained for each student in both contexts (circles) and the KA Model of Eq 1 using the coefficients obtained with the MLR approach (squares).

At last, we show in Fig 7 a comparison between the final grade for each student and the final knowledge of Eq 1 (KA model) with the coefficients obtained with the MLR approach. Again, the *K*_*f*_ obtained with the model behaves similarly to the data. It should be noted the similarity of the result obtained in Figs 5 and 6 with that shown in Fig 2 of [19]. In the present work, the adjustment of the weights that gave rise to both figures was carried out in a more appropriate way than in that paper, where the coefficients of each term were chosen exploratory.

## Discussion

In a previous work we proposed to describe the knowledge acquisition process as a dynamic quantity composed of several terms, where it was implicit that such a process was carried out in the classroom. But, what happens when the physical place where this complex socio-cultural construction takes place changes? What are the consequences of that specific educational context being taken from one day to the next? We seek to answer these questions by discussing how the terms of the knowledge acquisition model were modified, and which ones most directly influenced student performance during the transition to virtuality.

For that, we analyze the knowledge acquisition process in face-to-face and virtual contexts for a specific study case. Our investigation spanned two years and involved 173 students, observing the evolution of their learning process for each particular context.

Inspired by the work of Ref. [19], we wanted to assess whether the observed changes in academic performance can be understood from a model that incorporates the main factors that contribute to the knowledge acquisition process. The KA model is an analytical model based on data, which incorporates information from a series of surveys and whose results are contrasted with information on academic performance. The surveys were carried out 3 times during each semester and reflected the feelings of the students during their learning process that influenced their performance.

The raw data in Fig 1 show that the final grade of the students in both contexts presented differences. Specifically, the grades of the students with High-achievement (HA) were better in virtual context than in face-to-face context. The opposite is seen with Low-achieving students (LA), while the intermediate performance group (AA) did not show differences. In the results shown in [18], the performance of the students who used remote learning tools showed an improvement in the virtual context. We believe that this difference is due to the fact that having better resources positioned them in a privileged place with respect to the case studied in this work.

The results obtained in Fig 3 reinforce accepted ideas related to the importance of motivation in the learning process: the switch to virtual context caused a negative impact on the motivation of the entire student population, but was strongly reflected in the performance of LA students. This fact should alert the educational community and especially those responsible for building support mechanisms for the mental health of students. Furthermore, we observe that the new context generates a change in the way students interact with their peers and teachers. In particular, the HA students did not modify the interaction with the teachers (maintaining high values in both contexts) while they strengthened the study in groups in the virtual context, unlike the rest of the groups. For AA and LA students, interaction with teachers increased in the virtual context, and this result highlights the importance of the teacher’s role as a consultant and as fundamental support for students.

We also find that the structure of the network of contacts that is formed between peers in both contexts presents some common characteristics, as well as some interesting differences, as we saw in Fig 4. Among the first is that both networks have highly connected clusters, as well as a significant number of completely isolated students. The virtual context network, however, shows a feature not observed in the other network: the presence of individuals who interact with one or more students from different clusters. These individuals act as bridges between students who otherwise would not be connected. These structures could be reflecting a new form of relationship between students that occurs more easily in the virtual context. Nevertheless, we are aware that this analysis requires a more detailed investigation that is beyond the scope of this work with the data we currently have. On the other hand, it is also true that the virtual context made it possible to record that the interactions between the students go beyond what happens in the classroom space.

Related with the previous analysis is the fact that, although the equation in the KA model is linear, the term of peers can be interpreted as an effective version of a real non-linear interaction. This term in itself adds complexity to the model since group interaction does not obey “linear” rules. However, the simplification made in the KA model remains valid in light of the results obtained in [19] and are in line with the idea that the learning process is not limited to the interactive behavior of individual teachers and students, but should be understood in terms of collaborative behavior [29].

In order to find out the relevance of the factors that we included in the KA model, we used two different approaches: a standard Multiple Linear Regression Method and a Single Layer Perceptron, which is a particular type of Artificial Neural Network.

The results obtained with the neural network (Fig 5) indicate that in both contexts the weights are similar. This result also shows that the raw results adequately describe each context, since the data obtained in each situation reflect the particular reality that each group of students is going through.

Moreover, both approaches indicate a greater relevance of the term of interaction with teachers. We were able to collect information from the teachers to support this fact and the perception of the change in the interaction with the students was also commented on by them (see S2 File). The knowledge acquisition process comes hand in hand with the importance of the interaction with teachers, and the literalness of their presence in the accompaniment during learning. This result also confirms in some way the universality of the educational act.

The comparisons of Figs 6 and 7 between the raw data and the results obtained with the KA model indicate that the general behavior of individuals can be suitably described with Eq 1, which is simply the sum of the relative contributions of each of the proposed factors: personal motivation, interaction with peers and influence of teachers. The robustness of the coefficients obtained with the two approaches also indicates that the information collected in the surveys and observations was sufficient to construct an adequate representation of the process. We are aware that this simplification leaves out a huge number of variables that are integrated to give rise to the unique process that each person experiences. But we believe that the results obtained allow us to validate our choice of factors as the main contributions common to all individuals.

Now, we discuss some considerations on the scope and limitations of this work.

One is that we must not lose sight of the fact that the change in the specific physical context brought with it a change in the evaluation criteria. Actually, this aspect was addressed in the teacher interviews that we summarize in the (S2 File). As *K*_*f*_ is a hard data (the final grade obtained in the course), it would be more appropriate to build new models that consider these data in a more comprehensive way, taking into account the challenges that arose due to the change in this educational context.

Another important issue that is absent from the KA model is the personal context of the students and their available resources. The reason why it was not included is because we had no survey done on these topics in the face-to-face period, so it was not possible to compare both contexts. However, in the Supplementary Material (S2 File) we include additional information regarding this subject obtained from the surveys carried out in the virtual context. When asking the students for their feelings regarding confinement, the responses were varied but reluctance was reflected in more than half of the responses. This coincides with our observation about the lack of motivation (see Fig 3(b)). The emotional stress, widely discussed in this context, goes beyond the academic environment and it was an important characteristic that we tried to capture with our research. Moreover, we found some relevant differences between the students of the different groups, which could influence their performance. Among them, a third of the students belonging to the LA group said they had a poor Internet connection in contrast to the HA group in which this situation occurred for a sixth of the students. More importantly, 13% of students belonging to the LA group did not have a laptop computer and 30% did not have an adequate study space.

These results show how the pandemic has increased educational inequalities at the economic, technological, social and even emotional level of the actors in the educational process. The virtual context promoted a change in teaching and learning methodologies, but it also brought another great challenge that is still far from being resolved, namely access to resources for all students. Hence the importance of recognizing inequalities to make visible the urgent need to build university policies that improve this situation.

A final though has to do with the generalizability of our results. Although this study was done for a specific case, the main factors analyzed here (motivation, interaction with peers and teachers) are not isolated from the global scenario. The generalization of the KA model to other educational scenarios is not only possible but quite straightforward. It should be noted, however, that the part of our study referring to the virtual context was carried out during the first year of the pandemic, so the results obtained could be strongly influenced by the transition between both contexts. Nevertheless, we believe they are valuable in themselves and can serve to deepen the understanding of the complex process of learning.

## Supporting information

S1 File Survey data: Numerical values associated with the KA model. (XLSX)

S2 File Additional information obtained from student and teacher surveys. (PDF)

## References

[1] Driver R, Asoko H, Leach J, Scott P, Mortimer E. Constructing scientific knowledge in the classroom. Educational researcher. 1994;23(7):5–12. doi: 10.3102/0013189X023007005

[2] Anderson RM, Heesterbeek H, Klinkenberg D, Hollingsworth TD. How will country-based mitigation measures influence the course of the COVID-19 epidemic? The lancet. 2020;395(10228):931–934. doi: 10.1016/S0140-6736(20)30567-5 32164834 PMC7158572

[3] Wang X, Pasco RF, Du Z, Petty M, Fox SJ, Galvani AP, et al. Impact of social distancing measures on coronavirus disease healthcare demand, central Texas, USA. Emerging infectious diseases. 2020;26(10):2361. doi: 10.3201/eid2610.201702 32692648 PMC7510701

[4] Duque D, Morton DP, Singh B, Du Z, Pasco R, Meyers LA. COVID-19: How to relax social distancing if you must. medRxiv. 2020;29. doi: 10.1101/2020.11.26.20152520 32727898 PMC7443931

[5] Ferguson NM, Laydon D, Nedjati-Gilani G, Imai N, Ainslie K, Baguelin M, et al. Impact of non-pharmaceutical interventions (NPIs) to reduce COVID-19 mortality and healthcare demand. Imperial College COVID-19 Response Team. Imperial College COVID-19 Response Team. 2020; p. 20.

[6] Prem K, Liu Y, Russell TW, Kucharski AJ, Eggo RM, Davies N, et al. The effect of control strategies to reduce social mixing on outcomes of the COVID-19 epidemic in Wuhan, China: a modelling study. The Lancet Public Health. 2020;5(5):e261–e270. doi: 10.1016/S2468-2667(20)30073-6 32220655 PMC7158905

[7] Giordano G, Blanchini F, Bruno R, Colaneri P, Di Filippo A, Di Matteo A, et al. Modelling the COVID-19 epidemic and implementation of population-wide interventions in Italy. Nature medicine. 2020;26(6):855–860. doi: 10.1038/s41591-020-0883-7 32322102 PMC7175834

[8] Flaxman S, Mishra S, Gandy A, Unwin HJT, Mellan TA, Coupland H, et al. Estimating the effects of non-pharmaceutical interventions on COVID-19 in Europe. Nature. 2020;584(7820):257–261. doi: 10.1038/s41586-020-2405-7 32512579

[9] Sinha D, Tan P. Mathematical model and simulations of COVID-19 2020 outbreak in New York: Predictions and implications for control measures. Available at SSRN 3584911. 2020;.

[10] Block P, Hoffman M, Raabe IJ, Dowd JB, Rahal C, Kashyap R, et al. Social network-based distancing strategies to flatten the COVID-19 curve in a post-lockdown world. Nature Human Behaviour. 2020;4(6):588–596. doi: 10.1038/s41562-020-0898-6 32499576

[11] Sands D, Kormos L, Nowak J, Vaughan H, Voice A, Zochowski S. Moving teaching online during the COVID-19 pandemic. Europhysics News. 2020;51(4):30–32. doi: 10.1051/epn/2020406

[12] Schleicher A. The impact of COVID-19 on education insights from education at a glance 2020. Retrieved from oecd org website: https://www.oecd.org/education/the-impact-of-covid-19-on-education-insights-education-at-a-glance-2020.pdf. 2020;.

[13] Various. Policy Brief: Education during COVID-19 and beyond. United Nations; 2020.

[14] Di Pietro G, Biagi F, Costa P, Karpiński Z, Mazza J. The likely impact of COVID-19 on education: Reflections based on the existing literature and recent international datasets. vol. 30275. Publications Office of the European Union; 2020.

[15] Marinoni G, Van’t Land H, Jensen T. The impact of Covid-19 on higher education around the world. IAU Global Survey Report. 2020;.

[16] Rashid S, Yadav SS. Impact of Covid-19 pandemic on higher education and research. Indian Journal of Human Development. 2020;14(2):340–343. doi: 10.1177/0973703020946700

[17] Aristovnik A, Keržič D, Ravšelj D, Tomaževič N, Umek L. Impacts of the COVID-19 pandemic on life of higher education students: A global perspective. Sustainability. 2020;12(20):8438. doi: 10.3390/su12208438 PMC8634691 34869802

[18] Gonzalez T, De La Rubia MA, Hincz KP, Comas-Lopez M, Subirats L, Fort S, et al. Influence of COVID-19 confinement on students’ performance in higher education. PloS one. 2020;15(10):e0239490. doi: 10.1371/journal.pone.0239490 33035228 PMC7546684

[19] Velásquez-Rojas F, Laguna MF. The knowledge acquisition process from a complex system perspective: observations and models. Nonlinear Dynamics, Psychology, and Life Sciences. 2021;25(1):41–67. 33308389

[20] Browning MH, Larson LR, Sharaievska I, Rigolon A, McAnirlin O, Mullenbach L, et al. Psychological impacts from COVID-19 among university students: Risk factors across seven states in the United States. PloS one. 2021;16(1):e0245327. doi: 10.1371/journal.pone.0245327 33411812 PMC7790395

[21] Malecki CK, Elliot SN. Children’s social behaviors as predictors of academic achievement: A longitudinal analysis. School psychology quarterly. 2002;17(1):1. doi: 10.1521/scpq.17.1.1.19902

[22] Cunha F, Heckman J. The technology of skill formation. American Economic Review. 2007;97(2):31–47. doi: 10.1257/aer.97.2.31

[23] Web page of Engineering Faculty. UNLP; 2021. https://www.ing.unlp.edu.ar/.

[24] Bordogna CM, Albano EV. Theoretical description of teaching-learning processes: A multidisciplinary approach. Physical Review Letters. 2001;87(11):118701. doi: 10.1103/PhysRevLett.87.118701 11531550

[25] Rosenblatt F. The perceptron: a probabilistic model for information storage and organization in the brain. Psychological review. 1958;65(6):386. doi: 10.1037/h0042519 13602029

[26] Goodfellow I, Bengio Y, Courville A. Deep learning. MIT press; 2016.

[27] Chollet F, et al. Keras; 2015. https://github.com/fchollet/keras.

[28] Team R. R: A language and environment for statistical computing (R Version 4.1.0, R Foundation for Statistical Computing, Vienna, Austria, 2020); 2021.

[29] Stamovlasis D, Koopmans M. Complex Dynamical Systems in Education: Concepts, methods and applications; 2016.

