# Supplementary material for: Effects of the COVID-19 pandemic in higher education: A data driven analysis for the knowledge acquisition process
Source: PLoS One. 2022 Sep 7;17(9):e0274039. doi: 10.1371/journal.pone.0274039 (PMC9451099; doi:10.1371/journal.pone.0274039)
Supplement: S2 File — (PDF) [file pone.0274039.s002.pdf]

S2 FILE: Additional information obtained from students and teachers surveys

Supporting information file for the manuscript: Effects of the COVID-19 pandemic in higher education: a data driven analysis for the knowledge acquisition process, by F. Velásquez-Rojas, J.E. Fajardo, D. Zacharías and M.F. Laguna.

This file displays data collected during the virtual context that was not included in the KA model described by Eq. 1 of the manuscript. However, we consider that information about the resources that students have (workspace, technological equipment), the context in which they find themselves and how they perceive it, provide valuable and complementary information to understand their situation in a more comprehensive way. This information is presented in the first two sections, whereas the last one summarizes the main responses of an interview with the teachers of the students included in this study.

I. Student's personal context and their available resources

As mentioned in the manuscript, the surveys performed include questions to find out the personal or individual context and the resources that the students had in the stage of virtual learning. Below we detail the questions posed and present the information obtained for each group of students, classified according to the final grade K_f they obtained in the subject. Thus, the groups are made up of high-achieving students, HA ($8 \leq K_f \leq 10$), average-achieving students, AA ($6 < K_f < 8$) and low-achieving ones, LA ($4 \leq K_f \leq 6$). Students with a final grade lower than 4 are not included in this study.

A. Regarding the available resources, they were asked:

**How would you rate your internet connection?**			
	**HA**	**AA**	**LA**
**Good**	16 (84.2%)	31 (73.8%)	21 (67.7%)
**Bad**	3 (15.8%)	11 (26.2%)	10 (32.3%)

Note that approximately 1 in 3 students belonging to the LA group said they had a poor internet connection in contrast to the HA group in which this situation occurred for 1 in 6 students.

**From where do you connect to receive the information on the course (emails, theories, virtual sessions)?**			
	**HA**	**AA**	**LA**
**Desktop computer, notebook, laptop**	19 (100%)	39 (92.9%)	27 (87.1%)
**Cellphone, Tablet**		3 (7.1%)	4 (12.9%)

**Do you have physical space in the place where you live (or are you passing the confinement) to be able to study and do the activities of the Faculty?**			
	**HA**	**AA**	**LA**
**Yes**	17 (89.5%)	36 (85.7%)	21 (67.7%)
**Sometimes**	2 (10.5%)	6 (14.3%)	8 (25.8%)
**No**			2 (6.5%)

For the students who participated in our research, only in the group HA do we find that all students had a laptop/computer. Besides, having adequate study space was not a feature present in all cases, especially for the LA students.

According to the EU report[1], access to relevant digital learning resources (e.g. laptop/computer, broadband internet connection) is generally limited in students from disadvantaged backgrounds who find it difficult to have a suitable learning environment at home (for example, a quiet place to study or your own desk) during this virtual context. The report also states that "Several studies find that children's cognitive and socio-emotional skill levels are good predictors of later outcomes". We believe that recognizing inequalities is the first step to change them. Hence the need to build university policies to improve this situation.

B. Regarding the individual context and feelings, they were asked:

**Who accompanies you during confinement?**			
	**HA**	**AA**	**LA**
**Family**	18 (94.7%)	37 (88.1%)	27 (87.1%)
**Friends, partners, nobody, others**	1 (5.3%)	5 (11.9%)	4 (12.9%)

The same report cited above indicates that it is common that in better-off families the transition to the virtual learning environment is easier, since students have better conditions and more resources. Although most of the students are in a family environment, isolated cases can be decisive in this respect.

The previous question should be connected to the next one, since feelings about quarantine can be tempered by social and family relationships.

[1] Di Pietro G, Biagi F, Costa P, Karpinski Z, Mazza J., *The likely impact of COVID-19 on education: Reflections based on the existing literature and recent international datasets.* Vol. 30275. Publications Office of the European Union; 2020.

How do you feel you are facing the quarantine?			
	**HA**	**AA**	**LA**
**Good**	7 (36.8%)	18 (42.9%)	9 (29.0%)
**Not bad not good**	8 (42.1%)	19 (45.2%)	17 (54.9%)
**Bad**	4 (21.1%)	5 (11.9%)	5 (16.1%)

When asking the students for their feelings regarding confinement, the responses were varied and this is reflected in the lack of motivation observed in Fig. 3b of the manuscript. The emotional stress, widely discussed in this context, goes beyond the academic environment and taking it into account it was an important part that we wanted to capture in our proposal.

II. Student comments

In all the surveys carried out during the virtual context, we added an optional final question. We received more than 90 responses to this question (by 64 different students of which 44 were male and 20 female) and we retrieved the following information:

Q. Do you have a final comment?

- None of the students who answered the question say that interaction with their peers helped them. In fact, they do not speak of this as a strategy to facilitate their learning.
- Motivation (or lack thereof) is manifested in the answers but few in a direct way. 11 students say that virtuality discouraged them or made learning too difficult for them.
- The vast majority focus on interaction with teachers. Several propose improvements or changes in the strategies used by the chairs, but there is a general consensus on the effort and commitment that teachers are putting into carrying out the course, expressed in 33 responses.
- 8 students say they would have a better time in person vs. 3 who say that this virtual context worked for them.
- 3 students explicitly expressed difficulties due to lack of resources.
- Only 1 student says that the strategies deployed in virtuality should be maintained for colleagues who work and cannot attend in person.

III. Interview with teachers: an overview

The survey we present here was answered by 7 teachers (3 male, 4 female) from the sections that participated in this research. In all of the cases they had to develop each answer. We present here a summary of the responses and some outstanding phrases.

Q1. Regarding the face-to-face context, how did your way of evaluating in the virtual environment change?

The evaluations were virtual. Teachers indicated that they increased the difficulty or looked for atypical problems (other than those that were solved during the classes) to compensate for the fact that the students had all the material available at the time of the exam. In addition, in some cases they made follow-ups based on questionnaires.

Outstanding phrase: “To offset the impact of the extra difficulty of taking an online midterm, and the impossibility of daily face-to-face follow-up, optional weekly grading activities were implemented.These asynchronous activities tried to motivate the students to study the subject in a sustained way. Those students who passed the exam corresponding to the module, obtained an extra score proportional to the work done”.

Q2. In general, do you think the assessment / evaluation standards have been lowered?

4 answered yes, 3 no. One of the latter observed that by maintaining the previous standards, the performance of the students lowered.

Outstanding phrase: “We have had a high dropout rate, in which many factors are confluent according to an absolutely personal reading: academic overload, connectivity difficulties, lack of devices for an adequate connection, drastic habit changes in times of pandemic, in addition to economic, social and emotional factors that we are very far from being able to glimpse from our role as teachers”.

Q3. What strategies did you incorporate in your classes in the virtual context?

Synchronous hourly load reduction (from 8 to 6 hours a week). Doubt box (to plan the explanation reinforcements on this basis). PowerPoint presentations. Use of virtual platforms and their accessories (zoom, zoom board, google classroom for communications). Recorded theoretical classes accessible on YouTube Armed with videos with short classes (between 15 and 30 minutes). Free access to a lecture book with all the topics covered. To accompany the reading, we prepare guidelines for each chapter, offering a guide to what we want to highlight topic by topic. Synchronous encounters to address the difficulties arising from viewing the videos. Some chairs (not all) prepared optional self-assessment questionnaires for different topics.

Q4. Do you have a final comment?

A teacher comments on differences in the "level" of the students in both semesters (those in the 2nd semester are recurrent, so he assumes that they have a lower level). He observes a decrease in interest (measured by the number of inquiries made) in the 2nd semester. Another one comments that teachers have learned to teach virtual classes in a reasonable way. Not so the exams, which he qualifies as the most conflictive point of virtuality. Another professor comments that the forced experience of virtual work has left the concern of incorporating some of the tools already used in 2020 and incorporating new ones.

Outstanding phrase: “This approach has not been easy, it meant a lot of work, many hours a day to prepare our new material. Adapting to the new reality implied, among other things, that we had to learn to build new tools. This stage was also traveled with the support of our working groups. Teamwork was essential to face these new ways of teaching”.
